# Standardized cardiovascular magnetic resonance (CMR) protocols 2013 update

**DOI:** 10.1186/1532-429X-15-91

**Published:** 2013-10-08

**Authors:** Christopher M Kramer, Jörg Barkhausen, Scott D Flamm, Raymond J Kim, Eike Nagel

**Affiliations:** 1 Departments of Medicine and Radiology, University of Virginia Health System, Lee Street, Box 800170, Charlottesville, VA 22908, USA; 2 Department for Radiology, University Hospital Schleswig-Holstein, Lübeck, Germany; 3 Imaging Institute, and Heart and Vascular Institute, Cleveland Clinic, Cleveland, OH, USA; 4 Duke Cardiovascular Magnetic Resonance Center, Duke University Medical Center, Durham, NC, USA; 5 Division of Imaging Sciences, King’s College, London, United Kingdom

## Abstract

This document is an update to the 2008 publication of the Society for Cardiovascular Magnetic Resonance (SCMR) Board of Trustees Task Force on Standardized Protocols. Since the time of the original publication, 3 additional task forces (Reporting, Post-Processing, and Congenital Heart Disease) have published documents that should be referred to in conjunction with the present document. The section on general principles and techniques has been expanded as more of the techniques common to CMR have been standardized. There is still a great deal of development in the area of tissue characterization/mapping, so these protocols have been in general left as optional. The authors hope that this document continues to standardize and simplify the patient-based approach to clinical CMR. It will be updated at regular intervals as the field of CMR advances.

## Introduction

This document is an update to the 2008 publication of the Society for Cardiovascular Magnetic Resonance (SCMR) Board of Trustees Task Force on Standardized Protocols [1]. Since the time of the original publication, 3 additional task forces have published documents that should be referred to in conjunction with the present document. The first is the document on Reporting published by that SCMR Task Force in 2010 [2]. All references to reporting of the findings from the protocols listed have been removed from the present document as they are covered in the aforementioned document. In addition, all references to analysis methodologies have been removed from the current protocols document as they are covered in their entirety in the recent publication of the SCMR Task Force on Post-Processing [3]. All protocols relative to congenital heart disease have been removed, as a separate document has been recently published in regards to this topic [4].

The section on general principles and techniques has been expanded as more of the techniques common to CMR have been standardized. There is still a great deal of development in the area of tissue characterization/mapping, so these protocols have been in general left as optional. The authors hope that this document continues to standardize and simplify the patient-based approach to clinical CMR. It will be updated at regular intervals as the field of CMR advances.

## General Techniques

### Field Strength Considerations

1. CMR can be performed at different field strength. 1.5T systems are currently used for the majority of examinations.

2. CMR at 3T requires careful shimming and adjustment of the radiofrequency pulses to avoid artifacts.

3. As a result of improved signal-to-noise ratio (SNR), 3T is advantageous for first pass contrast-enhanced perfusion imaging. Furthermore tagging sequences and 4D flow techniques may benefit from 3T.

4. Steady-state free precession sequences, often the choice for cine imaging at 1.5T, have several challenges at 3T including increased dark banding artifacts, flow artifacts, and suboptimal flip angle choices because of specific absorption rate (SAR) restrictions.

5. Devices that have been tested at 1.5 T may not be safe at 3T: check specific information relating to MRI safety of devices at higher magnetic field strength.

### Stress agents

1. Dobutamine: typical maximum dose 40 ug/kg/min

2. Atropine: 0.25 mg fractions typical (maximal dose 2 mg)

3. Adenosine:140 μg/kg body weight/min, (consider an increase up to 210 μg/kg body weight/min depending on institutional and local norms if after 2–3 minutes, HR does not increase by 10 bpm and or blood pressure does not drop by >10 mm Hg)

4. Regadenoson: 0.4 mg single injection.

#### ***Contraindications***

Dobutamine

• Severe systemic arterial hypertension (≥ 220/120 mmHg)

• Unstable angina pectoris

• Significant aortic valve stenosis (Peak aortic valve gradient >50 mmHg or aortic valve area < 1 cm^2^)

• Complex cardiac arrhythmias including uncontrolled atrial fibrillation

• Hypertrophic obstructive cardiomyopathy

• Myocarditis, endocarditis, pericarditis

• Uncontrolled congestive heart failure

Atropine

• Narrow-angle glaucoma

• Myasthenia gravis

• Obstructive uropathy

• Obstructive gastrointestinal disorders

Adenosine or Regadenoson

• 2nd or 3rd degree atrioventricular (AV) block or sinus node dysfunction

• Systolic blood pressure less than 90 mm Hg

• Sinus bradycardia (heart rate<40 bpm)

• Active bronchoconstrictive or bronchospastic disease with regular use of inhalors

• Known hypersensitivity to adenosine or regadenoson

• (Side effects are described as less significant with regadenoson than with adenosine, however, the half life of regadenoson is longer)

#### ***Patient preparation***

1. Obtain informed consent for the stress test

2. To fully exert its effects patients should optimally refrain from the following medications for 12–24 hours prior to the examination due to potential of counteraction against stress agent.

Dobutamine: ß-blockers and nitrates.

Adenosine/regadenoson: caffeine (coffee, tea, caffeinated beverages or foods e.g. chocolate, caffeinated medications), theophylline, dipyridamole

Note: There is increasing data that the effects of caffeine and nicotine can be overcome by higher doses of adenosine as well as regadenoson.

1. Fasting is not mandatory, but is often advised because recognized adverse effects of stress drugs include nausea and vomiting, which may be problematic when lying supine in the restricted space of the magnet

2. Two intravenous lines should be available, one for gadolinium and one for adenosine, one in each arm. Preferential site of contrast infusion is antecubital. Blood pressure cuff should be used with care taken not to interfere with gadolinium or adenosine infusion. For regadenoson only one line is required.

#### ***Potential adverse effects***

Dobutamine at high doses may cause chest pain, palpitations. More severe complications are rare, including:

• infarction

• ventricular fibrillation

• sustained ventricular tachycardia

Adenosine and regadenoson may cause flushing, chest pain, palpitations, breathlessness. More severe side effects include

• transient heart block

• transient hypotension

• transient sinus tachycardia

• bronchospasm

### Stress equipment and safety

1. Monitoring equipment (blood pressure, electrocardiogram for monitoring of cardiac rhythm, intercom to communicate with patient)

2. Preparation and regular practice for rapid removal of the patient from the magnet

3. Emergency resuscitation policy in place

4. Defibrillator

5. Drugs for emergency treatment

a. Immediately at hand: ß-blocker (eg esmolol or metoprolol), nitroglycerin, aminophylline

a. In the emergency cart: full set of emergency drugs (including drugs such as: epinephrine, beta blockers, atropine, bronchodilators, antiarrhythmic drugs)

6. For dobutamine - on-line assessment of wall motion during image reconstruction performed immediately after image acquisition

### Gadolinium dosing module/safety

See Table 1.

**Table 1:** Contrast and chasing bolus doses and injection rates

**Indication**	**Contrast dose (mmol/kg body weight)**	**Injection rate**	**Saline chasing bolus**	**Injection rate**
**Perfusion**	0.05-0.1	3-7 mL/s	30 mL	3-7 mL/s
**Late gadolinium enhancement**	0.1-0.2		20 mL	
**Angiography (carotids, renals, aorta)**	0.1-0.2	2-3 mL/s	20 mL	2-3 mL/s
**Time-resolved angiography**	0.05	3-5 mL/s	30 mL	3-5 mL/s
**Peripheral angiography**	0.2	First 10 mL @ 1.5 mL/s, rest @ 0.4-0.8 mL/s	20 mL	0.4-0.8 mL/s

Notes:

1. Volumes and injection rates depend on scan duration: the given values are recommendations for typical scan times.

2. Injection rates are different for 1 molar contrast agents. As a general rule, divide the given injection rates by a factor of 2.

3. Contrast agents with higher relaxivity (e.g., gadobenate dimeglumine) require smaller doses.

4. Throughout the protocols, the term "gadolinium" refers to gadolinium chelates

Safety considerations:

1. The use of gadolinium contrast should be avoided in patients with stage 4 or 5 chronic kidney disease (estimated glomerular filtration rate <30 mL/min/1.73 m^2^), particularly for those on dialysis, as well as patients with acute renal failure and chronic liver disease, due to concerns regarding nephrogenic systemic fibrosis (NSF).

2. The dose of gadolinium contrast used should be as low as possible to achieve adequate image quality.

3. The risk of NSF depends in part upon the gadolinium chelate used. Use institutional, regional, or national guidelines to guide choice of agent in patients with renal dysfunction.

4. If no alternative is available in dialysis patients such that gadolinium must be used, dialysis should be performed as per institutional, regional or national guidelines.

### Left ventricular structure and function module

1. Scout imaging – transaxial, coronal, sagittal

2. Transaxial (8–10 mm) set of steady state free precession (SSFP) or fast spin echo images through the chest.

3. Scout to line up short axis images – cine acquisitions are preferable to single shot as long axis motion and inflow should be visualized

a. Vertical long axis prescribed orthogonal to transaxial scouts aligned through the apex and center of the mitral valve

a. Horizontal long axis aligned orthogonal to the vertical long axis, passing through the apex and center of the mitral valve

4. SSFP is the method of choice for cine imaging because it provides high SNR and excellent contrast between myocardium and blood pool

a. At 3T, SSFP cine images may be compromised by artifact and spoiled gradient-echo sequences can be considered as an alternative

a. Strategies to reduce or move banding artifact include shimming, reducing the TR, and adjusting the RF frequency (frequency 'scout’ sequence can be helpful for this)

5. Steady state free precession short axis cine images, from the mitral valve plane through the apex. The basal most short axis slice should be located immediately on the myocardial side of the atrioventricular junction at end-diastole prescribed from the previously acquired long axis cines.

a. Slice thickness 6–8 mm, with 2–4 mm interslice gaps to equal 10 mm.

a. Temporal resolution ≤45 ms between phases

a. Parallel imaging used as available

6. Steady state free precession long axis cine images

a. The 4 chamber long axis is prescribed from the vertical long axis through the apex and center of the mitral and tricuspid valves. This can be cross-checked on basal short axis cines, using the costophrenic angle (margin) of the RV free wall.

a. Vertical long axis, prescribed from the scout already acquired

a. LV outflow tract (LVOT) long axis, passing through the apex, the center of the mitral valve and aligned with the center of LVOT to aortic valve, as seen on a basal short axis cine.

a. Optional – a set of more than 3 rotational long axis views can be obtained.

### Right ventricular structure and function

1. Right ventricular (RV) short axis views can be obtained in a similar fashion to the LV structure and function module. If the short axis is used for quantification, it is important to place the basal short axis slice immediately on the myocardial side of the right ventricle and to take extra care to exclude appropriate amounts of atrial volume from at least one basal slice at end systole.

2. Transaxial stack of cines covering the RV should be considered for RV volumetry.

3. Long axis images should include an RV vertical long axis view aligned with tricuspid inflow and a RV outflow tract view (sagittal or oblique sagittal plane through the pulmonary valve).

### First pass perfusion module

1. Scout imaging as per LV structure and function module

2. Saturation-recovery imaging with gradient echo-echo planar (GRE-EPI) hybrid, GRE, or SSFP readout

3. Short-axis view imaging (at least 3 slices per heart beat)

a. For ischemia evaluation, must obtain data every heart beat

a. Slice thickness 8 mm

a. Parallel imaging, if available

a. In-plane resolution, ~ <3 mm

a. Readout temporal resolution ~ 100 – 125 ms or shorter as available

a. Contrast is given (0.05 – 0.1 mmol/kg, 3–7 mL/s) followed by at least 30 ml saline flush (3–7 mL/s)

a. Breathhold starts during early phases of contrast infusion before contrast reaches the LV cavity.

a. Image for 40–50 heart beats by which time contrast has passed through the LV myocardium

### Late gadolinium enhancement module

1. Pulse sequences:

a. 2D segmented inversion recovery GRE or SSFP, Phase-Sensitive Inversion-Recovery (PSIR), and 3D sequences are preferred in appropriate patients with satisfactory breathholding ability and if signal-to-noise is sufficient.

b. Single-shot imaging (SSFP readout) performed as backup for patients with irregular heart rhythm, and/or difficulty breath holding.

2. Need at least 10 minute wait after gadolinium injection (for gadolinium-chelate dosing see Table 1). Note - The delay may be shorter than 10 minutes if lower doses are used as blood pool signal falls below that of late enhanced myocardium. Images are to be acquired during diastolic stand-still.

3. Same views as for cine imaging (short- and long-axis views)

4. Slice thickness, same as for cine imaging

5. In-plane resolution, ~1.4-1.8 mm

6. Acquisition duration per R-R interval below 200 ms, but should be less in the setting of tachycardia to avoid image blurring.

7. Inversion time set to null normal myocardium. Alternatively, a PSIR sequence can be used, requiring less frequent adjustment of the TI.

8. Read-out is usually every other heart beat, but should be modified to every heart beat in the setting of bradycardia, and every third heart beat in the setting of tachycardia or arrhythmia.

### Adenosine/Regadenoson stress perfusion CMR

1. LV structure and function module (alternatively this can be performed between stress and rest perfusion, although performance immediately after gadolinium infusion may reduce the contrast of the blood-endocardium interface)

2. Adenosine stress perfusion imaging (at least 3 minute infusion of 140 ug/kg body weight/min, optional up to 210 ug/kg body weight/min). Option – initial adenosine infusion may be performed with the patient outside the bore of the magnet.

a. First pass perfusion module

b. During last minute of adenosine, gadolinium is injected

c. After imaging for 40–50 heart beats by which time gadolinium has passed through the LV myocardium, adenosine is stopped.

d. Continuous ECG monitoring and BP measured at baseline, during infusion, and for at least 2 minutes post-infusion of adenosine.

3. Alternatively: Regadenoson stress perfusion imaging (bolus injection of 0.4 mg).

a. First pass perfusion module

b. Approximately 2 minutes after regadenoson injection, inject gadolinium

c. Image for 40–50 heart beats by which time gadolinium has passed through the LV myocardium

d. Continuous ECG monitoring and BP measured at baseline and every other minute for at least 6 minutes post injection of regadenoson.

4. Rest Perfusion

a. Need at least 10 minute wait for gadolinium to wash out from stress perfusion imaging. During this period stress images can be reviewed, cine imaging can be completed (e.g. long-axis views), valvular evaluation can be performed, etc.

b. Perfusion imaging repeated without adenosine/regadenoson using same dose of gadolinium (Note: flow may not have returned to baseline at 10 minutes after regadenoson)

c. If stress images are normal and free of artifacts, rest perfusion can be eliminated. Additional gadolinium may be given as needed for late gadolinium enhancement (for a total of 0.1 – 0.2 mmol/kg)

5. Late Gadolinium Enhancement module

a. Need to wait at least 5 minutes after rest perfusion if performed

6. Optional - Full quantification

a. Consider using a dual bolus approach to eliminate effect of nonlinearity between contrast agent concentration and signal intensity. This requires injection of a diluted pre-bolus in a standardized fashion.

a. Consider using a dual contrast sequence. Similarly to the dual bolus approach this corrects for non-linearity of signal intensity and contrast agent concentration without additional contrast dilution and injection but requires specific scanner software that may not be available on all scanners

a. Consider adding proton density images before the contrast injection. This can be used as baseline correction for full quantification but requires specific scanner software that may not be available on all scanners.

### Dobutamine stress CMR

1. LV structure and function module

2. Dobutamine stimulation

a. Increase the dobutamine in increments of 10 μg/kg body weight/minute every 3 minutes starting at 10 μg/kg body weight/minute until target heart rate [85% x (220-age)] reached.

a. Add atropine in small incremental doses, if heart rate response is inadequate (See 1.2, Stress agents)

a. Repeat 3 short axis and 3 long axis cine views during each increment

a. Continuous ECG monitoring and BP measured during each stage.

a. View cine loops online as they are being acquired.

a. Adapt the SSFP cine sequence to optimize temporal resolution as needed as the heart rate increases.

a. Stop test for new wall motion abnormality, serious side effect, or achievement of peak heart rate.

### Flow Module

1. Usually performed as part of other cardiovascular protocols. Available scout images can be used. Best if vessel of interest is depicted in two orientations or MRA can be reformatted on the scanner (e.g., additional bSSFP, CE-MRA, or T2 black blood scouts are helpful)

2. Sequence: one-direction (“through-plane”) motion-encoded cine gradient echo sequences are typically applied

3. For optimal results, the imaging plane should be

a) centered in the vessel of interest

b) aligned orthogonally to the expected main blood flow direction in two spatial directions

c) centered in the iso-center of the magnet

4. Imaging parameters: slice thickness 5-8 mm; in-plane resolution at least 1/10th of the vessel diameter. Velocity encoding sensitivity (V_enc_) has to be adapted to the expected velocities – after each scan, phase difference images have to be checked for aliasing. If aliasing is present, V_enc_ settings need to be adapted accordingly. If available, a velocity scout may allow optimal setting of the V_enc_.

5. Acquired time frames on the order of 20–30 suffice for clinical routine. For read-out, k-space segmentation over multiple heart beats can be used within limits of breath holding capabilities. Navigator-based non-breathhold techniques can be applied to improve the temporal or spatial resolution if necessary.

6. Echo time (TE) should be set to minimal, particularly when stenoses are imaged

### Advanced Tissue Characterization Module

The area of tissue characterization is a rapidly developing field and the pulse sequences available on different vendor platforms vary significantly. Thus, the modules listed below are general guidelines only as there is no standardization yet in this arena. Normal values should be developed at individual or partner institutions using similar platforms and pulse sequences.

1. T2-W imaging (optional)

a. Black blood T2-W STIR (Short Tau Inversion Recovery)

i. Potential pitfalls – bright signal in areas of low flow, signal dropout due to motion

a. Bright blood T2-W sequences

i. T2-prepared single-shot SSFP sequence

i. Turbo spin echo-steady state free precession hybrid is an alternative

i. Potential pitfall – bright signal may obscure endocardial border

a. T2 mapping (optional)

i. T2-prepared single-shot SSFP sequence acquired with different T2 prep time.

i. Motion correction as needed

2. T1 mapping (optional)

a. Look Locker imaging (MOLLI or SHMOLLI or equivalent)

i. performed prior to contrast and at 2–4 time points post contrast bolus

i. Alternatively, constant infusion of contrast can be used rather than bolus

### T2* Module

1. Performed to assess cardiac iron deposition in disease entities such as thalassemia major. Images typically acquired in concert with LV function imaging. If T2* images are acquired as part of a contrast-enhanced evaluation for cardiomyopathy, the T2* images should be obtained prior to contrast administration.

2. The pulse sequence is a single breathhold, gradient-echo, multi-echo scan with a series of 6–9 echo times beginning at ~2 ms and extending to ~18 ms, with each echo iteratively spaced by ~2 ms. A delay time of 0 ms after the R wave typically is used.

Optional - In patients with severe iron deposition a pulse sequence with shorter echo spacing could be helpful to accurately determine T2* values: a series of 6–9 echo times beginning at ~1 ms and extending to ~12 ms, with each echo iteratively spaced by ~1 ms.

3. A single mid-ventricular short-axis image is acquired.

4. Slice thickness of 8–10 mm; In-plane resolution, ~1.6-3.0 mm

5. (Optional) An imaging sequence similar to the above, though non-ECG-gated, is acquired in the axial orientation through the mid portion of the liver to evaluate hepatic iron deposition. The absence of ECG-gating will allow for closer spacing of iteratively advanced echo times, and therefore a greater number of echoes will be acquired.

## Disease specific protocols –

### Ischemic heart disease

#### ***Acute MI or acute coronary syndromes***

1. LV structure and function module

2. Optional - Advanced tissue characterization module

3. Optional - First pass perfusion module (only at rest). Consider stress if culprit vessel has already been revascularized.

4. To look for microvascular obstruction, consider repeat first pass perfusion sequence or early gadolinium enhancement, i.e. within the first 1–3 minutes after contrast infusion

5. Late gadolinium enhancement module

#### ***Chronic ischemic heart disease and viability***

1. LV structure and function module

2. Optional - Advanced tissue characterization module

3. Optional - low dose dobutamine with 5–10 minute infusion of 10 μg/kg/min of dobutamine to assess contractile reserve as improvement in wall thickening

4. Optional - adenosine stress-rest perfusion or high dose dobutamine functional imaging (see stress protocols for more details) to determine the presence of inducible perfusion deficits or wall motion abnormalities

5. Late Gadolinium Enhancement module

### Angiography

#### ***Peripheral MRA***

1. Peripheral vascular coil, or combination of coils, as available. Venous compression cuffs (placed on the thighs, and inflated to sub-diastolic pressure) are helpful, if available.

2. Transaxial, low-resolution, vessel scouting with time-of-flight MRA or SSFP.

3. Gadolinium timing

a. Option 1 -Transaxial test bolus at level of distal abdominal aorta. 2 mL injection of gadolinium, followed by 20 mL saline. Determine time to peak enhancement following injection using a single-shot bolus tracking sequence.

a. Option 2 – Bolus trigger technique to time start of scan

4. Stepping-table, gadolinium-enhanced MRA performed in the coronal projection from the mid abdominal aorta to the feet.

a. Two volumetric acquisitions – one pre-contrast (for subtraction) and one during contrast administration.

a. Gadolinium injected in 2 phases to minimize venous contamination followed by saline bolus. See Table 1

a. Slice thickness 1–1.5 mm; acquired spatial resolution in-plane 0.8-1.5 mm.

a. Slices – typically 60–100, as needed to accommodate vessels of interest.

a. Volumes obtained of abdomen/pelvis and thighs may be coarser spatial resolution (larger vessels), while those of the legs preferably are sub-millimeter spatial resolution. The former acquisitions typically require 15–20 seconds, while the leg acquisition may take 60–90 seconds for increased spatial resolution. Elliptical centric k-space acquisition is advantageous for the legs. If available, time-resolved acquisitions are preferred for the legs.

a. Parallel acquisition recommended (multichannel surface coil needed)

Alternative: dual injection protocol

1. Single dose of gadolinium: time-resolved MRA of the calf and foot vessels

2. Single dose of gadolinium: abdominal and thigh vessels

Alternative: Non-contrast MRA technique

1. Two general potential approaches are used (and there are others):

1. “Fresh Blood Imaging” where two ECG-triggered 3D fast-spin-echo sequences are performed with the first gated to systole and the second to diastole. Subtraction of the systolic image from the diastolic image set results in an arterial-only image dataset. This is more amenable to larger volumes and greater z-axis acquisitions

a. Slice thickness ~2 mm; acquired spatial resolution in-plane 0.6-0.8 mm.

a. Slices – typically 40, as needed to accommodate vessels of interest.

a. Parallel acquisition recommended (multichannel surface coil needed)

2. 3D SSFP with an inversion preparation pulse, which provides suppression of background tissue, and with an appropriate TI, allows for the inflow of arterial blood from outside the IR prepared volume and into the region of interest providing high arterial signal. This is more suited toward smaller volume acquisitions

a. Volume acquired: ~340 × 300 × 70; acquired spatial resolution ~1.3 × 1.3 × 1.4.

b. Parallel acquisition recommended (multichannel surface coil needed)

#### ***Thoracic aortic MRA***

1. Localizer, 3 orientations

2. Half-fourier single shot fast spin echo or SSFP (one breathhold, entire thorax) Transaxial orientation.

3. Transaxial T1-weighted fast spin echo through aorta (for intramural hematoma, dissection)

4. SSFP cine imaging in parasagittal plane parallel to and along midline of aorta Option – use 3-point piloting

5. Evaluate aortic valve as per valvular protocol

6. Contrast timing

a. Option 1 -Transaxial test bolus at level of distal abdominal aorta. 2 ml injection of gadolinium, followed by 20 ml saline. Determine time to peak enhancement following injection.

a. Option 2 – Bolus triggering technique to time start of scan

a. Option 3 – Rapid multiphase 3D acquisitions without timing

7. 3D gadolinium enhanced MRA (0.1-0.2 mmol/kg) (optional – ECG-gated acquisition)

a. Use spatial resolution of at least 1-1.5 mm

a. Parallel acquisition if available

a. At least 2 acquisitions after contrast injection

8. Optional - transaxial T2-weighted gradient echo or T1-weighted gradient-echo post-contrast for aortitis

9. Optional – see section 3.2.1 above (Peripheral MRA) for noncontrast MRA techniques

### Anomalous coronary artery evaluation

1. LV structure and function module to look for wall motion abnormalities

a. Add repeat horizontal long axis with high temporal resolution sequence (≤ 20 ms per phase) to accurately determine quiescent period of RCA

2. Navigator-gated, 3D, free-breathing, MRA sequence:

d. Parallel acquisition preferred

d. Navigator placed over the right hemi-diaphragm.

d. Optional – gadolinium contrast to increase vessel conspicuity

e. Optional –

f. Breathhold techniques if poor image quality or navigators unavailable or of poor quality

f. T2-prepared sequence may be useful

d. Transaxial slices spanning from level of proximal main pulmonary artery down to the middle of the right atrium (entire cardiac coverage if desired). Slice thickness 1–1.5 mm; acquired spatial resolution in-plane of 1.0 mm or less.

d. Slices – typically 50–80, as needed to encompass vessels of interest.

d. Adjust trigger delay and acquisition window according to observed quiescent coronary period.

#### ***Pulmonary vein evaluation – pre- and post-ablation***

1. LV structure and function module

2. Breathhold 3D contrast-enhanced MRA performed in the coronal projection encompassing the pulmonary veins and left atrium (greater anterior coverage if breathholding permits)

a. Optional – oblique plane centering the pulmonary veins can reduce slab thickness and therefore breath hold time but will lead to less coverage of the left atrium

a. Optional - ECG-gating. When patient has irregular rhythm, readout should be synchronized with systole (i.e. no trigger delay)

a. 2–3 volumetric acquisitions – one pre-contrast (for subtraction), one during the first pass of contrast administration, one (optional) after contrast administration

a. Gadolinium (0.1-0.2 mmol/kg) injected at 2–3 mL/s

a. Slice thickness 1–2 mm; acquired spatial resolution in-plane 1–1.5 mm.

a. Slices – typically 60–80, as needed to encompass region of interest.

a. Parallel acquisition used as available

3. Optional – through plane phase contrast flow analysis through each pulmonary vein.

#### ***Coronary venography***

1. Navigator-gated, 3D, free-breathing, inversion prepared MRA sequence:

a. Transaxial slices spanning from level of proximal main pulmonary artery down to the middle of the right atrium (entire cardiac coverage if desired). Slice thickness 1–1.5 mm; acquired spatial resolution in-plane of 1.0 mm or less.

b. Slices – typically 110–130, as needed to encompass vessels of interest.

c. Adjust trigger delay and acquisition window according to end-systole (= maximal vein diameter) or alternative quiescent period during mid-diastole, typical acquisition duration per heart beat 60-90 ms.

d. Parallel acquisition preferred

e. Fat suppression

f. Navigator placed over the right hemi-diaphragm.

g. No T2 preparation prepulse, but Inversion prepulse (determine inversion time before scanning using TI-scout or Look-Locker)

h. Perform after administration of contrast agent

### Other

#### ***Nonischemic LV cardiomyopathies including myocarditis***

1. LV structure and function module

2. Optional - Advanced tissue characterization module

3. Optional – for myocarditis only

Early gadolinium enhancement ratio - T1-weighted imaging before and after gadolinium with measurement of ratio of change in signal intensity in myocardium to change in skeletal muscle.

4. Late Gadolinium Enhancement module

5. Optional - adenosine stress-rest perfusion imaging or high dose dobutamine stress functional imaging (see stress protocols for methods and contraindications) to comment on the presence or absence of ischemia since a mixed cardiomyopathy may be present

#### ***Hypertrophic Cardiomyopathy***

1. LV structure and function module

2. LV outflow tract flow phase imaging including SSFP cine in a 3-chamber view examining for turbulence and systolic anterior motion of the mitral valve or chordae, and phase velocity measurements for gradient if present

3. Optional – consider advanced tissue characterization module

4. Optional – consider vasodilator stress perfusion module

5. Late gadolinium enhancement module

#### ***Arrhythmogenic right ventricular cardiomyopathy***

1. LV structure and function module – consider 5–6 mm slice thickness

2. Transaxial or oblique transaxial SSFP cine images (slice thickness 5–6 mm) covering the RV including RVOT. An RV vertical long axis view aligned with tricuspid inflow is recommended.

3. Optional sequences

a. Selected transaxial or oblique transaxial black blood images (double inversion recovery T1-weighted fast spin echo).

a. Repeat same geometry with fat suppression

a. Late gadolinium enhancement module in same orientations as above. Consider T1 nulling for RV.

#### ***Valvular disease***

Patients with artificial valves can safely undergo CMR at 1.5 and 3 Tesla. The force exerted by the beating heart is many-fold higher than the force exerted by the magnetic field.

1. LV structure and function module.

a. Use horizontal long axis to look for valve anatomy and turbulence of the mitral and tricuspid valve.

a. Use LVOT view for mitral and aortic valve.

a. Use vertical long axis for mitral valve.

a. Coronal view for aortic valve

a. Additional views (RV long axis, RV-outflow tract as needed).

2. Specific issues

a. Valve morphology assessment with SSFP cine in the plane of the valve in question. Care must be taken to optimize the level and angle of imaging

a. Note – if planimetry of a stenotic valve is to be attempted, a contiguous or slightly overlapping stack of high resolution cines transecting the line of the jet and moving from orifice level to immediately downstream is recommended. Planimetry is most likely to be valid where the cross section of the orifice, or rather of the jet, is clearly delineated. This may not always be the case due to fragmented or oblique jet flow.

a. Gradient echo or hybrid gradient echo/echo planar imaging may visualize regurgitant jets with a higher sensitivity (for qualitative purposes only).

a. In mitral or tricuspid regurgitation, a contiguous stack of 5 mm cines is recommended aligned with the direction of inflow and transecting the principal line of coaptation, moving from the more superior commissure to the inferior. The orientation can be that of the LVOT plane for the mitral and transaxial for the tricuspid. Such a stack enables assessment of tethering, prolapse, or regurgitation through the scallops of both mitral leafletes.

a. Adapt velocity encoding to actual velocity (using lowest velocity without aliasing).

a. Use lowest TE possible for high velocity jet flows.

#### ***Pericardial disease***

1. LV structure and function module

2. T1 or T2-weighted fast spin echo images

a. 2–3 representative long axis images and 3–4 representative short axis images to measure pericardial thickness (normal ≤3 mm)

a. If pericardial cyst is suspected, refer to masses protocol

3. Optional - If regions of thickened pericardium noted - T1-weighted gradient echo myocardial tagged cine sequences to demonstrate presence or absence of epicardial/pericardial slippage (2–3 long axis images and 1–2 short axis images)

4. Real-time imaging during dynamic breathing maneuvers is valuable for evaluation of ventricular interdependence

a. Mid-ventricular short-axis plane is preferred

b. Cine imaging temporal resolution is preferably below 60 ms

c. Patients are instructed to breathe deeply in and out and the total imaging period should be at least 2 complete respiratory cycles

d. Abnormal septal motion (early diastolic septal flattening or inversion) during onset of inspiration is consistent with a constrictive physiology

5. Late Gadolinium Enhancement module

a. Acquisition with and without fat saturation is helpful to distinguish pericardial inflammation from epicardial or pericardial fat

#### ***Cardiac and paracardiac masses, including thrombi***

1. LV structure and function module

2. T1 weighted fast spin echo - slices through the mass and surrounding structures (number of slices depends on size of the mass)

3. T2 weighted fast spin echo with fat suppression (optional - without fat suppression) - through the mass and surrounding structures as above. See nonischemic cardiomyopathies for sequence details.

4. First pass perfusion module with slices through mass

5. Repeat T1 weighted turbo spin echo with fat suppression

6. (optional) Repeat selected steady state free precession cine images post-contrast

7. Late gadolinium enhancement module

a. Images with the TI set to null thrombus (approximately 500–550 ms at 1.5T, 850–900 ms at 3T) will help differentiate thrombus from tumor as well as delineate thrombus surrounding or associated with tumor

a. Serial imaging can help distinguish hypoperfused tumor necrotic core from thrombus

## Competing interests

Christopher Kramer MD - Research support from Siemens Healthcare, Novartis. Consultant for Synarc, St. Jude Medical.

Jorg Barkhausen MD - none.

Scott Flamm MD - Institutional research support from Siemens Healthcare and Philips Healthcare. Advisory Board for Bayer Healthcare, TeraRecon and Circle Cardiovascular.

Raymond Kim MD - Inventor on a US patent owned by Northwestern University concerning delayed contrast-enhanced MRI to diagnose myocardial viability and infarction, Co-founder, HeartIT, LLC, Educational grant, Siemens Medical Solutions, Consultant: Bayer, Velomedix, St. Jude Medical.

Eike Nagel MD - Research support from Philips Healthcare and Bayer Healthcare.

## Authors’ contributions

CMK: wrote protocols, edited protocols, edited manuscript, corresponding author. JB: wrote protocols, edited protocols, edited manuscript. SDF: wrote protocols, edited protocols, edited manuscript. RJK: wrote protocols, edited protocols, edited manuscript. EN: wrote protocols, edited protocols, edited manuscript. All authors read and approved the final manuscript.
